# The Impact of Aerobic Exercise on HDL Quantity and Quality: A Narrative Review

**DOI:** 10.3390/ijms24054653

**Published:** 2023-02-28

**Authors:** Beata Franczyk, Anna Gluba-Brzózka, Aleksandra Ciałkowska-Rysz, Janusz Ławiński, Jacek Rysz

**Affiliations:** 1 Department of Nephrology, Hypertension and Family Medicine, Medical University of Lodz, 90-549 Lodz, Poland; 2 Palliative Medicine Unit, Oncology Department, Medical University of Lodz, 90-549 Lodz, Poland; 3 Department of Urology, Institute of Medical Sciences, Medical College of Rzeszow University, 35-055 Rzeszow, Poland

**Keywords:** aerobic exercise, HDL levels, HDL structure, HDL functioning, training intensity, training duration

## Abstract

High-density lipoproteins comprise roughly 25–30% of the circulating proteins involved in the transport of lipids in circulation. These particles differ in size and lipid composition. Recent evidence suggests that the quality of HDL particles (which depends on shape, size and the composition of proteins and lipids determining HDL functionality) may be more important than their quantity. The functionality of HDL is mirrored by its cholesterol efflux activity, as well as its antioxidant (including the protection of LDL against oxidation), anti-inflammatory and antithrombotic properties. The results of many studies and meta-analyses imply the beneficial impact of aerobic exercise on HDL-C levels. Physical activity was found to be usually associated with an increase in HDL cholesterol and a decrease in LDL cholesterol and triglycerides. Exercise, apart from inducing quantitative alterations in serum lipids, exerts a beneficial impact on HDL particle maturation, composition and functionality. The Physical Activity Guidelines Advisory Committee Report underlined the importance of establishing a program recommending exercises that enable attainment of maximal advantage at the lowest level of risk. The aim of this manuscript is to review the impact of different types of aerobic exercise (various intensities and durations) on the level and quality of HDL.

## 1. Introduction

High-density lipoproteins (HDL) comprise roughly 25–30% of the circulating proteins involved in the transport of lipids in circulation [1]. These particles have a complex structure; they are of different sizes and lipid compositions. Both the quantity and quality of HDL appear to be influenced by aging, disease states, exposure to some environmental factors (e.g., pollutants) and pathogens, smoking and diet [2]. Therefore, the level of HDL cholesterol fluctuates during our lifetime [3,4]. Moreover, according to studies, a sedentary lifestyle is related to low HDL levels, irrespective of age and sex [5,6]. Low HDL levels are usually accompanied by high triglyceride (TG) levels, insulin resistance and abdominal obesity [7]. However, in individuals with hypoalphalipoproteinemia, relatively normal total lipids and decreased HDL cholesterol (HDL-C) are observed [8]. In the Health, Risk Factors, Exercise Training and Genetics (HERITAGE) Family Study, men with isolated HDL also had very low mean plasma cholesterol, low-density lipoprotein (LDL), apolipoprotein B (apoB) and apoA-I levels [9]. They were not obese, and their abdominal fat accumulation did not differ from normolipidemic individuals. HDL cholesterol, apart from being involved in reverse transport, exerts various effects, including anti-inflammatory, antioxidative, antidiabetic, antithrombotic, and many activities [10]. The results of studies have demonstrated that isolated low HDL cholesterol may translate into a greater risk of coronary heart disease (CHD) [11]. Reduced plasma levels of HDL cholesterol are a common abnormality reported in patients with CHD [12]. The results of randomized, controlled drug trials have demonstrated that a pharmacological increase in HDL levels does not translate into ameliorated cardiovascular disease (CVD) outcomes [13,14,15]. Therefore, later interventions are focused on the improvement of the quality and functionality of HDL, not only its levels. It has been suggested that exercise, especially aerobic exercise, may have the potential to improve the atheroprotective functions of HDL [16]. The results of studies and meta-analyses have suggested the beneficial impact of aerobic exercise on HDL-C levels [2,5]. Regular exercise and a healthy diet are considered to be crucial for the maintenance of a normal lipid profile and subsequent reduction in cardiovascular risk [16]. Available evidence indicates that acute and chronic aerobic exercise may increase plasma levels of HDL cholesterol in a dose-dependent manner [17,18].

The aim of this manuscript is to review the impact of different types of aerobic exercise (various intensities and durations) on the level and quality of HDL. We conducted a PubMed search to identify articles suitable for inclusion in this narrative review. We did not perform a systematic review. Terms that were searched for included “Aerobic exercise”, “HDL structure”, “HDL levels”, “HDL functioning”, “chronic exercise”, “acute exercise”, “low-moderate intensity training” and “high intensity training”.

## 2. Structure and Functions of HDL Cholesterol

HDL particles are very heterogenous and can be subdivided into several subclasses with distinct functionalities [19]. The division of HDL into subclasses depends on the method of separation. In the literature, HDL is most frequently subdivided into two principal HDL subclasses via ultracentrifugation, i.e., HDL2 (larger particles) and HDL3 (smaller particles) [20,21,22]. HDL2 can be further divided into HDL2a and HDL2b. These subfractions contain higher amounts of cholesterol, and their role in reverse cholesterol transport is more pronounced. In turn, HDL3 is a more nascent form of HDL that contains less cholesterol and shows higher density compared to HDL2 [19,22]. With the use of the Lipoprint system, it is possible to obtain as many as 10 HDL subfractions that belong to 3 subclasses: large, intermediate and small.

HDL particles possess atheroprotective properties since they participate in reverse cholesterol transport [16]. Reverse cholesterol transport involves the transfer of peripheral cholesterol to HDL particles and its transportation to the liver for excretion. The initial step of this process, macrophage-specific cholesterol efflux, appears to be crucial for HDL-related protection against atherosclerosis [16]. ATP binding cassette A-1 receptor (ABCA-1) is involved in the taking up of cholesterol from macrophages in the artery wall by nascent HDL [23]. In the early stage of cholesterol efflux, lipid-free apoA-I attaches to cholesterol, forming discoidal HDL [2]. In the next stages, more free cholesterol becomes bound, and lecithin:cholesterol acyltransferase (LCAT) catalyzes its esterification to cholesteryl ester (CE), which leads to the formation of a core of spherical HDL. Following the binding with phospholipid (PL), free cholesterol (FC) and apoA-I form disc-shaped HDL particles, which subsequently undergo growth and maturation. The transformation of discoidal HDL into spherical HDL is associated with greater cholesterol efflux and the formation of cholesteryl ester. The process of HDL maturation involves the accumulation of cholesteryl ester. During this step, smaller HDL3 particles grow to form larger HDL2 particles enriched with cholesterol with a larger particle size [2]. The role of reverse cholesterol transport in cardiovascular protection has been confirmed in numerous animal and human studies. Apart from its role in reverse cholesterol efflux, HDL cholesterol also possesses other important physiological properties, such as anti-inflammatory, antioxidative and antithrombotic effects, which are partly mediated by bound enzymes [24]. The functionality of HDL is partly associated with the presence of various protein and lipid compounds. Apolipoprotein A-I is a vital HDL protein, the content of which may reach 70% and which appears to be responsible for the anti-inflammatory and antioxidant properties of HDL [2,25]. This protein is also involved in cholesterol efflux activity [26]. ApoA-I contributes to HDL quality; therefore, its diminished content is considered a risk factor for cardiovascular events [2]. ApoA-II is the second most crucial HDL apolipoprotein. Its increased serum levels have been suggested to be related to combined hyperlipidemia, higher indices of atherogenic lipoproteins and atherosclerosis development [26,27]. The results of studies demonstrated that higher content of apoA-II in HDL was associated with paraoxonase displacement from the particle and decreased HDL antioxidant properties [28]. Moreover, apoA-II could affect the interaction of HDL with scavenger receptor-B-I (SR-BI) and impair HDL functioning via the involvement of apoA-I displacement in a reconstituted HDL [29]. The enrichment of HDL particles in apoC-III may also cause functional and structural impairment of HDL, leading to the acquisition of more atherogenic properties by HDL. Higher content of apoC-III is associated with the senescence-related truncation of apoA-I and greater HDL glycation [30]. The results of in vitro studies indicate that higher apoC-III and decreased content of apoA-I in HDL may be related to the formation of dysfunctional HDL [31,32]. Furthermore, apoC-III was suggested to induce alternative inflammasome activation, leading to organ damage and subsequently enhanced mortality, especially in patients with chronic kidney disease and acute myocardial infarction [33]. Human serum amyloid A (SAA) is another particle associated with HDL in the plasma, especially dysfunctional HDL. The roles of lipid-free and HDL-associated forms of SAA are different [34]. Lipid-free SAA plays a role in innate immunity and the repair of tissues. It has been found to be involved in inflammatory cytokine induction, the chemotaxis of leukocytes and the upregulation of genes regulating extracellular matrix remodeling [35,36,37]. However, these actions appear to be mostly abrogated if the SAA is attached to HDL [38]. The enhanced incorporation of this acute-phase reactant has been demonstrated to indicate ongoing systemic inflammation [39]. SAA-enriched HDL is susceptible to releasing lipid-free apoA-I, thereby promoting the formation of poor-quality HDL [40]. Dullaart et al. [41] demonstrated that the antioxidative properties of HDL are inversely correlated with the level of circulating SAA in patients with metabolic syndrome. Therefore, it seems that higher SAA concentrations may hamper the antioxidative activity of HDL [39]. Lipids contained in HDL particles, for example, triglycerides and cholesterol, also affect the quality of HDL [2]. Higher content of cholesterol in HDL is associated with larger particle size, while increased content of TG results in the formation of smaller particles and impaired HDL functionality, which leads to disturbed cholesterol transport and efflux. The results of studies have revealed that decreased content of cholesterol and higher content of TG in HDL particles increase the risk of metabolic syndrome. Highly functional HDL particles are large, with a distinct round shape, and they contain a high amount of apoA-I [2]. Several enzymes are associated with HDL particles, including an esterase paraoxonase 1 (PON1), platelet-activating factor acetylhydrolase (PAF-AH) and LCAT. Paraoxonase 1 is involved in the antioxidative activities of HDL particles, while PAF-AH participates in antithrombotic actions [42,43]. Paraoxonase-displaying lactonase and ester hydrolase activity can degrade lipid peroxides in LDL and hinder viral infections [2,44]. In a healthy state, native HDL protects LDL from oxidation since it neutralizes free radicals and reactive oxygen species (ROS), but it is also involved in the transport of oxidized LDL (oxLDL) to the liver for excretion. The process of atherosclerosis is associated with the presence of oxidative stress and the subsequent formation of oxidized LDL cholesterol. OxLDLs are potent triggers of atherosclerosis, and their presence is associated with an increased risk of cardiovascular diseases and cerebrovascular diseases [45,46]. HDL cholesterol hampers the oxidation of LDL via the metabolization of lipid hydroperoxides, thereby limiting their accumulation on low-density lipoproteins [47]. The results of studies have demonstrated that HDL is also capable of taking up lipid peroxides and transporting them to the liver for excretion [48]. The vasoprotective activity of HDL is primarily associated with the stimulation of endothelial production of nitric oxide, which exerts vasodilatory effects [39]. Some disease states are associated with the presence of dysfunctional HDL with altered properties. HDL quality and functionality can be impaired by many stressors, including aging, smoking, pollutants, infection and unhealthy food habits [2]. Dysfunctional HDL contains lower amounts of cholesterol; it is also enriched with TG, SAA and apoC-III, and the displacement of apoA-I is observed. These changes result in the alteration of HDL particle morphology, with particles becoming smaller in size and taking on an ambiguous shape due to exposure to glycation stress, oxidative stress, smoking, etc. The results of studies have demonstrated that generally, females tend to have higher plasma HDL-C and lower LDL-C and TG levels than males [49,50,51]. However, it appears that HDL mean particle size is larger in women (≥85 Å) than in men (<85 Å). Williams et al. [21] observed increased concentrations of HDL3b in post-menopausal women, as well as considerably higher HDL3c and HDL3b and significantly lower HDL2b and HDL2a levels in adult men than boys (<18 years). The levels of HDL3c and HDL3b are higher in adult than women, while the levels of HDL2b, HDL2a and larger-diameter HDL3a particles are lower in males. These results may indicate the impact of sex hormones on the determination of HDL levels. It has been suggested that the association between hormones, plasma HDL-C concentration and lipoprotein particles size is more complex and also involves visceral adipose tissue [50]. Both plasma concentrations of HDL and HDL size appear to be regulated by plasma triglyceride concentrations, lipase activities, insulin sensitivity and abdominal fat via their effect on the apoA-I fractional catabolic rate (FCR) [52]. Females were found to have lower FCR of apo A-I and apo A-II than men. FCR of apo A-I is the principal metabolic mechanism responsible for increased HDL cholesterol, and its low values are related to a lipid-rich HDL fraction [53]. The impact of hormones (estradiol) on increased HDL and apoA-I levels was confirmed in a study of a cohort of young transgender individuals (phenotypical males treated with puberty blockers followed by estradiol) [54]. The estradiol-related increase in HDL level was found to be dose-dependent and chromosome-independent, which implies that compared to atherogenic lipoproteins, HDL may be more sensitive to fluctuation of hormones levels at a young age. This study also indicated the influence of testosterone on increased very low-density lipoprotein (VLDL) levels in trans men (young phenotypical females treated with puberty blockers followed by testosterone).

The presence of obesity, diabetes mellitus and metabolic syndrome has been demonstrated to affect the lipoprotein profile. Obese individuals show a reduction in plasma HDL-C levels, as well as an increase in TG [55,56]. In obese individuals, the contents of apoA-I, cholesterol and phospholipid levels in HDL are decreased, while levels of serum amyloid are increased. This translates into a shift towards smaller HDL3 subclasses and reduced HDL2 and antioxidative capacity. The level of the HDL3 subfraction is strongly correlated with lecithin cholesterol acyltransferase (LCAT) activity. Such alterations were not observed in overweight females [55]. However, in overweight women, only TG content in HDL was increased. Stadler et al. [55] reported significant alterations in the activity of cholesteryl ester transfer protein (CETP) (involved in conversion of lipid-poor pre-β particles to HDL3) and LCAT and modified HDL composition. In obese women, the activity of cholesterol ester transfer protein (CETP) enzyme involved in HDL metabolism was reported to be increased, in agreement with the fact that adipose tissue synthesizes large amounts of CETP [57,58]. In obese women, LCAT activity and protein levels also appear to be significantly increased and correlate with reduced antioxidative capacity of HDL [55,59]. A similar relationship was demonstrated in diabetic females [55]. The increase in LCAT activity in obesity and obesity-associated low-grade inflammation may represent a compensatory mechanism [60,61]. According to Stadler et al. [55], enhanced activity of LCAT in obese females translates into increased formation of cholesteryl esters in HDL. Successive CETP-mediated transfer to triglyceride-rich lipoproteins in exchange for triglycerides leads to higher triglyceride content in HDL, accelerated HDL hydrolysis by hepatic and lipoprotein lipases and, eventually, to the formation of smaller, denser HDL3 particles, as well as decreased HDL2 cholesterol levels [62,63]. Davidson et al. [64] observed that obesity partly explained variability in HDL at the subspecies levels. This finding was confirmed in a study in which weight loss surgery reversed the atherogenic HDL profile previously observed in obese patients. Within one year of the surgery, an increase in larger HDL particles and a loss of small HDL particles were reported [65]. However, this effect could also be associated with the impact of surgery on gut physiology, which could affect HDL metabolism [66].

The results of studies revealed that diabetic females showed significantly higher plasma levels of TC, TG, LDL-C, HDL-C, non-HDL cholesterol, lipoprotein (a) and apolipoprotein B [67,68,69]. LCAT production and its activity also appear to be worse in women with type 2 diabetes compared with men [70]. Alterations in the HDL profile in patients with metabolic disease mainly involve an increase in small particles and a decrease in large HDL particles, which suggests an impact on HDL metabolism [64,71,72]. Enhanced lipid transfer or catabolism of large HDL particles in patients with metabolic disease may explain the prevalence of smaller HDL particles. Mendivil et al. [73] suggested that metabolic syndrome may not affect HDL metabolism but the production of larger particles and their release into circulation.

An increasing amount of evidence suggests that various types of exercises exert a beneficial impact on the lipid profile.

## 3. Aerobic Exercise

Exercise is a common approach used to treat obesity, as well as to decrease the risk of non-communicable disease development [74]. Exercise training has been found to be involved in epigenetic regulation [75]. The results of many studies have indicated that exercise training interventions decrease body fat content and blood pressure (BP) and improve the lipid profile, thereby reducing cardiovascular risk [24,76,77]. Moreover, exercise was found to ameliorate glycemic control in patients with type 2 diabetes mellitus, improve insulin sensitivity and resistance and exert protective effects against the development of Parkinson’s disease, multiple sclerosis, lung diseases, etc. [78,79,80,81]. Apart from being beneficial for physical health and the aging process, regular physical activity is also important for mental health [78].

Aerobic exercise (also known as endurance exercise) is based on cardiorespiratory endurance training, which involves the use of large muscles for long durations, such as jogging, cycling, swimming, walking, using an elliptical trainer, skiing, rowing, running and using an upper-body ergometer (a piece of equipment that provides a cardiovascular workout that targets the upper body only) [82,83,84]. Aerobic exercise has been demonstrated to influence blood lipid metabolism since it raises HDL-C by increasing lipoprotein lipase (LPL) concentration and activity in skeletal muscles [85]. Moreover, such types of activity accelerate lipid transfer, decomposition and excretion and reduces fasting or postprandial TG [84,86]. Apart from the aforementioned changes, it also lowers total cholesterol (TC) levels and serum low-density lipoprotein cholesterol (LDL-C) [87,88]. According to Physical Activity Guidelines for Americans (2nd edition) [89], to gain health benefit, moderate-intensity aerobic training should be practiced from 150 to 300 min a week; however, this time is shorter in the case of vigorous-intensity aerobic physical activity training (75 to 150 min a week). Argani et al. [90] suggested that even 30 min of exercise per day is sufficient to boost HDL cholesterol levels in diabetic patients. Exercise interventions have been suggested to favour the shift towards larger HDL subclasses via mechanisms that are independent of changes in body composition [91,92,93]. Ferguson et al. [94] observed that 8–14 weeks of aerobic exercise resulted in a reduction in fasting TG level of 4–37% and a rise in HDL-C concentration of 4–18%.

The results of studies have suggested that the responses of females and males to various types of physical activity may differ [95,96,97]. It has been observed that women benefit more from low-to-moderate-intensity aerobic exercise, while men require more intense activity to obtain advantageous effects. This finding is associated with differences in the structure and functioning of their bodies. For example, the cardiovascular system of men allows them to participate in more vigorous exercise, which is reflected by higher VO_2_max values, greater lung function, lower heart rates and higher red blood cell counts [96,97]. Moreover, during exercise, males use higher amounts of carbohydrates and proteins to produce energy, while women use more lipids [95].

## 4. Impact of Aerobic Exercise on HDL Levels

The results of studies have demonstrated that regular aerobic exercise can increase plasma levels of HDL and reduce the risk of CHD among active and fit persons [9,98,99,100]. However, the exercise training stimulus must be sufficient. It has been proposed that exercise-related rises in HDL levels may be due to the consequent loss of fat and body mass [9]. Couillard et al. [9] suggested that a standardized exercise training program is associated with specific favorable alterations in lipoprotein profile in men with low HDL and high TG. Such training, apart from increasing apoA-I and HDL cholesterol levels, also diminishes plasma total cholesterol, TG, LDL and apoB levels. The results of the Inter99 study including a total of 4039 men and women aged 33–64 years demonstrated a significant relationship between physical activity and beneficial change in total cholesterol (*p* = 0.006), LDL cholesterol (*p* = 0.007), triglycerides (*p* = 0.02) and HDL cholesterol (*p* = 0.01) [101]. However, Couillard et al. [9] reported that exercise was not able to increase plasma HDL in a patient with low levels of this lipoprotein. Similar results were obtained by Zmuda et al. [102], who also failed to observe the beneficial impact of training on HDL levels in subjects with low baseline HDL cholesterol. The authors suggested that the inability to ameliorate TG metabolism was the underlying cause of the observed effect. This hypothesis seems plausible since Couillard et al. [9] demonstrated that only in patients with low HDL accompanied by high TG levels was a rise in HDL observed following participation in a training program, as well as a reduction in TG. Moreover, they implied that the favorable impact of aerobic exercise on HDL levels in individuals with low levels of this lipoprotein is beyond plasma lipoprotein levels, insulin sensitivity and body mass control [9]. Patients with isolated low HDL levels were found to be less responsive to endurance training. In another study, the greatest improvement in HDL levels related to exercise was reported in men with high initial HDL cholesterol levels [103]. However, the authors ascribed the beneficial impact on HDL levels to weight loss and the subjects’ running mileage during the trial. An incremental maximal treadmill run to exhaustion significantly (24%) raised oxidized HDL lipids and reduced oxidized LDL lipids (by 19%), which translated into a 55% increase in the oxidized HDL/oxidized LDL lipids ratio [104]. These levels remained elevated after 15 min of recovery. The observed training-related effects could be the ascribed to greater transfer of lipid peroxides to HDL for reverse transport and their enhanced clearance. Numerous studies have confirmed that acute or chronic prolonged endurance exercise performed at low to moderate intensities (50–70% of the maximal aerobic capacity VO_2_max) increases HDL levels as a result of enhanced lipid/lipoprotein metabolic activity [9,105,106,107]. Short but high-intensity exercise appears to have less impact on lipid utilization, and it exerts hardly any effect on lipid levels [105,107,108]. Some controlled studies have demonstrated that even a single session of exercise at an intensity of 50–80% VO_2_max could induce beneficial effects in the form of increasing serum HDL cholesterol (4–43%) and decreasing serum triglycerides (3–15%), which appear 18–24 h after exercise and are maintained for up to 72 h [109]. A marathon run (one session of prolonged heavy training) was found to reduce LDL cholesterol by 4–38%. Banz et al. [110] compared the effects of aerobic and resistance training regimens in individuals with metabolic syndrome. After 10 weeks of exercise, no significant changes in TC, LDL cholesterol or TG were found in either group. However, HDL cholesterol increased by 13% (from 29.8 to 33.7 mg/dL, *p* < 0.05) but only in the aerobic training group. Some studies have found benefits of aquatic exercise, which appeared to be equally effective in improving lipid and lipoprotein levels as standard exercise [111]. Another study demonstrated an improvement in the total HDL cholesterol ratio only (a reduction from 3.41 to 2.92, *p* < 0.05) following 12 weeks of training (150 min of exercise weekly at 65% of VO_2_max) [112]. In this study, no effects on lipid profile were observed following an intense interval running protocol (40 min/week). The authors suggested that the training volume but not the training intensity was the main factor influencing the lipid profile [112]. However, Wood et al. [113] showed that high-intensity interval training (HIIT) was more effective at increasing HDL-C levels than moderate-intensity continuous training (MICT) in younger adults. Appropriate training volume is sufficient to induce changes in fat mass that are necessary to modify the lipid profile in a favorable manner. Some studies have found that even shorter interventions induce effects, providing that the training volume is high enough [83]. In turn, King et al. [114] implied that not only the exercise volume is important but also time, since it appears that older adults require a longer time to respond to exercise with a change in HDL-C levels. Other researchers suggested that older men and women may need a different training mode to fully benefit and to experience an increase in HDL-C; however, this issue awaits further studies [95,115]. The results of a large population study of older men and women that assessed the effect of 5 years of various exercise intensities on HDL-C appear to confirm this thesis. After 5 years, men involved in HIIT experienced a smaller reduction in HDL-C compared with the control group and MICT. However, no differences were observed in women. Changes in HDL-C correlated only with VO_2_ peak in both men and women [116].

Moreover, the impact on HDL appears to be more pronounced when aerobic exercise raises during continuous effort [83]. This thesis was confirmed in a study of participants subjected to a 6-month aerobic exercise training program in which intensity was increased from 50% to 85% of maximum aerobic power for 20–60 min three times weekly [117]. Such training was associated with a statistically significant reduction in total the cholesterol:HDL cholesterol ratio (−0.3, *p* < 0.001) and total cholesterol (−0.3 mmol/L, *p* < 0.001). In another 16-week study, training three times per week at 70–75% HR_max_ for 30 min for the first 8 weeks, progressing to four times weekly at 85% HRmax for 45 min, caused a significant decrease in plasma TG (from 1.4 to 1.2 mmol/L, *p* < 0.05) and an increase in HDL cholesterol (from 1.4 to 1.8 mmol/L, *p* < 0.05) [118]. Such intense exercise was also associated with a 13% diminished body fat percentage (*p* < 0.05), which implies that it triggered an additional metabolic response.

A study of the effects of growing volume and intensity of aerobic exercise in sedentary overweight participants with mild-to-moderate dyslipidemia demonstrated that the combination of high-intensity and high-volume training (jogging for 20 miles/week at an intensity of 65–80% of VO_2_ peak) for 8 months led to the improvement of the majority of lipid parameters, including an increase in HDL cholesterol (44.3 to 48.6 mg/dL, *p* < 0.05) and a reduction in LDL cholesterol (from 130.1 to 128.2 mg/dL, *p* < 0.05) and triglycerides (from 166.9 to 138.5 mg/dL, *p* < 0.05) [119]. The observed effects were more pronounced compared to high-intensity/low-volume aerobic exercise (jogging for 12 miles/week at an intensity of 65–80% VO_2_ peak) and moderate-intensity/low-volume exercise (walking for 12 miles/week at an intensity of 40–55% VO_2_ peak). The reduction in lipids was found to depend on total energy expenditure and intensity. Naharudin et al. [107], who analyzed the impact of a series of high-intensity exercise sessions (cycling at 90% VO_2_peak for 8 min) combined with a 40% energy-deficit diet (ED group) found a considerable increase in HDL level of 13.74% after 10 days accompanied by weight loss. These effects were not observed in the normal diet group of physically active collegiate males [107]. Obtained results imply that high-intensity exercise training sessions combined with energy deprivation could ameliorate plasma HDL levels in healthy males. Studies of the impact of regular exercise on plasma concentrations of HDL have provided conflicting results probably resulting from the use of different interventions, various types of patients and concomitant therapies (e.g., diet and medications) [17,91,120].

Various dietary factors, including the consumption of polyunsaturated, monounsaturated and saturated fat instead of dietary carbohydrates, were demonstrated to contribute to an increase in HDL-C by 7–12% [121]. Very low-carbohydrate diets (<10% or 50 g/d of carbohydrates) increased HDL-C (by 11%), LDL-C and TC; reduced TG; and contributed to weight loss compared to low-fat diets [122]. In turn, added sugars (except for highly glycemic carbohydrates) were demonstrated to reduce HDL-C. Increased HDL-C (9.2%) levels, independent of alterations in other lipids, were observed after alcohol consumption. The consumption of omega-3 fatty acids, following a Mediterranean diet and diet-related weight loss were also found to increase HDL-C (~4–5%) [121].

Prolonged aerobic exercise of moderate intensity is recommended as an optimal option for individuals who previously led a sedentary life and those who did not exercise. The exact mechanisms of the beneficial impact of exercise on the lipid profile remains unclear. It has been suggested that exercise boosts the ability of skeletal muscles to utilize lipids, not glycogen, consequently decreasing lipid levels [123]. It appears that this phenomenon is associated with an increase in the enzyme involved in ester transfer to HDL cholesterol—LCAT [124]. Indeed, higher levels of LCAT have been observed following exercise training [125]. A study of the impact of four different single exercise sessions on lipids, lipoproteins and lipoprotein lipase revealed that 1100 kcal of energy expenditure during a single exercise session is necessary to significantly enhance lipoprotein lipase activity and produce increases in HDL cholesterol [94]. However, the studies assessing exercise-related lipoprotein lipase activity provide inconsistent data [83,85].

**Figure 1:**
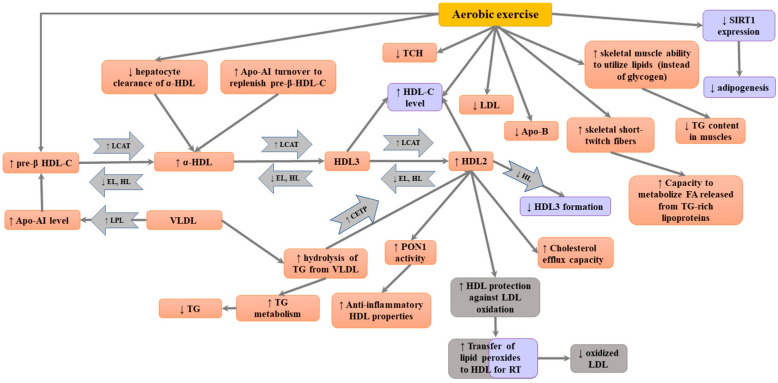
Possible mechanisms of the impact of exercise on HDL level and quality. Grey—effects of acute exercise; violet—effect of chronic exercise; orange—effects of exercise in general. apoA-I, apolipoprotein AI; CETP, cholesteryl ester transfer protein; FA, fatty acid; HDL, high-density lipoprotein; HTGL, hepatic triglyceride lipase; LCAT, lecithin:cholesterol acyltransferase; LDL, low-density lipoprotein; PON1, paraoxonase 1; RT, reverse transport; SIRT-1, sirtuin 1; TC, total cholesterol; TG, triglycerides.

The Consensus Committee (Panel) that met in 2000 reviewed data from 51 papers (including 28 randomized controlled trials (RCT) and a total of 4700 individuals) describing the impact of physical activity interventions on the lipid profile [109]. They suggested that physical activity is usually associated with a mean increase in HDL cholesterol by 4.6%, irrespective of gender, resulting in a decrease in LDL cholesterol of −3.7% and a decrease in triglycerides of 5%. According to the Panel, moderate-to-high-intensity exercise exerts beneficial effects on blood lipid and lipoprotein levels. In their analysis, the level of total cholesterol usually remained unaltered; however, the ratio of HDL to LDL cholesterol was improved. The experts suggested that aerobic exercise might have a stronger impact on triglycerides and LDL cholesterol compared to moderate physical activity [109]. They considered the evidence concerning acute, favorable changes in HDL cholesterol, serum triglycerides and LDL cholesterol as strong [109]. The Physical Activity Guidelines Advisory Committee Report underlined the importance of establishing a program, recommending exercises that enable the attainment of maximal advantage at the lowest level of risk [126]. According to a review by the European Society of Cardiology (ESC), there are no conclusive data concerning the amount of exercise required to improve the lipid profile and reduce cardiovascular risk in normolipidemic subjects and hyperlipidemic patients [127]. Possible mechanisms of the impact of exercise on HDL level and quality are summarized on Figure 1.

## 5. Impact of Aerobic Exercise on HDL Composition and Function

Recently, it has been revealed that the quality of HDL particles may be more important than their quantity. The term “HDL quality” refers to specific features of HDL, including size, shape and the composition of proteins and lipids determining HDL functionality [2]. As mentioned above, HDL particles contain cholesterol, triglycerides and phospholipids, as well as bound apolipoproteins and enzymes. The functionality of HDL mirrors its cholesterol efflux activity, as well as its antioxidant (including the protection of LDL against oxidation), anti-inflammatory and antithrombotic properties [2,128]. The lack of significant exercise-related changes in total HDL levels may be associated with favorable effects on HDL-C particle subfractions (higher levels of HDL2-C and lower levels of HDL3-C), which are not reflected by the concentration of total HDL-C [129].

Recent evidence suggests that exercise, apart from inducing quantitative alterations in serum lipids, exerts a beneficial impact on HDL particle maturation, composition and functionality [39]. Exercise training was found to trigger changes in HDL subclasses, especially an increase in large HDL subclasses, independently of body composition modifications [24,78,91,92].

In men, exercise training was demonstrated to promote higher HDL-C concentrations and HDL particle number that was mediated by the rise in HDL2b-C [22]. In women, a shift in HDL-C subfractions was reported. The authors concluded that exercise training translates into more healthy HDL subspecies distribution, without a change in total HDL-C concentration in overweight and obese women. Sarzynski et al. [91] also demonstrated that aerobic exercise can increase plasma levels of large HDL particles. Moreover, the results of the Health, Risk Factors, Exercise Training and Genetics (HERITAGE) Family Study indicate that a standardized exercise training program can increase HDL2 cholesterol and apoA-I in men, indicating the impact of exercise on both the density and number of HDL particles [9]. In contrast, the results of another study including 109 healthy subjects revealed that long-term physical activity was associated with a significant increase in HDL quantity in individuals who did not exercise previously; however, such training did not affect the levels of SAA and surfactant protein B (SPB) (which were used as markers reflecting the quality of HDL) [39]. However, long-term physical activity increased apoA-I levels by up to 11% and HDL quantity by up to 10%. Based on the assessment of SAA and SPB biomarkers, the authors concluded that long-term physical activity failed to change HDL quality [39]. Lipoprotein lipases have been suggested to play a role in the increase in HDL-C levels in response to exercise. The lipolysis of surface components in TG-rich lipoproteins followed by their fusion with HDL3 contributes to the enlargement of HDL particles and the consequent increase in HDL2 [130]. Moreover, aerobic training increases the percentage of skeletal slow-twitch fibers, which show greater capacity to metabolize fatty acids released by lipoprotein lipase from TG-rich lipoproteins [131].

The impact of exercise on HD functionality is more visible when HDL properties, not the levels of its subfractions, are analyzed. A study assessing the impact of six exercise interventions differing in amount and/or intensity on three different indices of cholesterol efflux capacity (CEC) revealed that only high amounts of aerobic exercise were capable of improving radiolabeled CEC [132]. Prediabetic individuals participating in the STRRIDE-PD study displayed a significant increase in CEC (+6.2%) after 6 months of a high amount and intense aerobic exercise in comparison to a low amount and the moderate-intensity group (−8.4%), as well as a high amount and the moderate-intensity group (−4.2%), independent of age, race, sex, BMI and the baseline CEC. The highest exercise dose was associated with significantly greater CEC (non-ATP-binding cassette transporter A1 (ABCA1)-mediated) compared to the control group in the E-MECHANIC study. However, in both the aforementioned studies, aerobic exercise failed to affect HDL–apoA-I exchange. These results suggest that the beneficial impact on HDL function may only occur after exceeding the exercise intensity threshold.

Acute exercise training was demonstrated to increase levels of nascent (pre-β) HDL-C without increasing either HDL-C or apolipoprotein A1 levels [133]. Pre-β HDL-C particles are generated from α-HDL-C when cholesterol esters are transferred to receptors of VLDL cholesterol or SR-BI; this observation may suggest that acute exercise stimulates the pre-β HDL-C formation rate via accelerated α-HDL-C particle utilization rather than de novo synthesis of apolipoprotein A1 [134]. Moreover, studies have demonstrated that the activity of cholesteryl acyl transferase, which takes part in the conversion of pre-β HDL-C to α-HDL-C, is increased immediately after acute exercise [135]. Reduced activity of cholesterol ester transfer protein translates into higher levels of HDL-C since it increases the concentration of cholesterol ester [136]. The increased activity of cholesterol ester transfer protein was suggested to trigger HDL-C turnover and stimulate the formation of pre-β HDL-C. The activity of hepatic lipase is associated with the formation of smaller, denser HDL-C and LDL-C particles via the hydrolysis of TG and phospholipids [137]. Prolonged exercise training was found to reduce its activity [138].

An increase in pre-β HDL-C levels without an increase in apolipoprotein A1 levels implies that training may inhibit hepatocyte clearance of α-HDL-C, promoting faster turnover of apolipoprotein A1, which enables replenishment of pre-β HDL-C [133].

A study assessing the early effects of short-term moderate-intensity exercise training (training for 3 months on bicycle ergometers) on HDL cholesterol plasma concentration and functionality in patients with metabolic syndrome (MS) failed to reveal any impact in terms of HDL plasma levels [92]. This type of exercise only reduced plasma triglycerides. However, such training exerted an impact on HDL functional characteristics. The training was associated with compositional changes in the smallest HDL subfractions, as well as increased HDL antioxidative capacity and paraoxonase-1 activity [92]. Moreover, 5% higher LDL resistance to oxidation in the presence of the HDL-2a and -3b subfractions was observed after 3 months of supervised training. Another study reported that a 4-month supervised exercise program resulted in greater HDL3 protection against LDL oxidation in diabetic patients. Interestingly, such an effect was not observed in healthy individuals participating in this study [93]. Enhanced lipid peroxide transport function of HDL was also reported in other studies. Endurance exercise training was found to improve HDL-dependent lipid peroxide transport function in a 6-month randomized controlled trial in sedentary women [139]. Another study revealed that aerobic training (wrestling and running) was associated with 1.3-fold higher HDL-C levels in Olympic athletes compared to anaerobic exercise performed by elite athletes, as well as with increased apoA-I content and paraoxonase activity and the prevalence of larger HDL particles [140]. This finding indicates that, apart from the impact on HDL levels, aerobic exercise may also influence HDL composition and functionality.

Moreover, Sang et al. evaluated the impact of 10 weeks of walk/run training on the anti-inflammatory properties of HDL in metabolic syndrome patients [141]. They demonstrated a greater ability of isolated HDL3 to reduce tumor necrosis factor α (TNF-α)-induced vascular cell adhesion molecule 1 (VCAM-1) expression and monocyte adhesion, which mirrored increased protection of endothelial cells against injury. The shift in HDL properties towards more anti-inflammatory (assessed on the basis of the HDL inflammatory index) was also reported in overweight or obese men with metabolic syndrome factors on a 3-week exercise intervention and residential diet [142]. The impact of a 21-day exercise intervention (plus diet) on HDL anti-inflammatory properties was also demonstrated in obese men [92].

In South African women, obesity was found to be related to higher levels of small HDL subfractions and lower levels of large subfractions compared to individuals with normal weight [143]. A study of the impact of 12-week exercise training on HDL functionality in obese black South African women revealed a reduction in small (potentially atherogenic) HDL subclasses, as well as diminished activity of PON and serum expression of PAF-AH, but no effect on HDL cholesterol level [24]. Since the alterations in HDL subfractions remained visible after adjustment for BMI and Waist-Hip Ratio (WHR), the authors suggested that they were potentially mediated by the stimulus of the exercise intervention. However, other studies have reported an increase in PON activity and HDL antioxidant function following exercise [92,93]. In Korean women, high-intensity aerobic training was associated with improvement not only in HDL but also LDL properties [144]. In these individuals, apoA-I content in HDL2 and HDL3 was increased, while apoA-II in HDL2 was reduced. HDL particles were of greater size and distinct shape and showed higher cholesterol content. Improved HDL function translated into lower levels of oxidated LDL cholesterol [144]. According to some authors, even intermittent training or several short exercises can affect lipoprotein and lipid changes; however, it appears that profound changes are associated with intense exercise sessions [145,146]. The mechanism of exercise-induced alterations in HDL functional characteristics remains not well defined. These changes are plausibly related to the molecular composition of HDL particles since the presence of specific protein and lipid components is associated with biological functions of HDL [147,148]. Some studies have suggested that the positive impact of exercise training on oxidative stress and inflammation contributes to changes in HDL subfractions [149,150]. Other studies revealed that aerobic exercise increases lipoprotein lipase, an enzyme that is vital for the formation of HDL [151,152]. Irrespective of the mechanism involved, exercise training is associated with beneficial changes in HDL, as well as other lipoproteins. Therefore, controlled and appropriately adjusted physical training is recommended to keep healthy. The summary of aforementioned studies is presented in Table 1.

## 6. Conclusions

The presented data suggest the favorable effects of aerobic exercise training on lipid and lipoprotein profiles. The impact of aerobic exercise on HDL function depends on several factors, including exercise type, intensity and duration, as well as participant characteristics (age, race, body mass, baseline HDL levels, diet, medications, etc.); therefore, studies provide sometimes conflicting results. Regular physical activity improves vascular function (e.g., endothelium-dependent vasodilation) via the effect on vascular structure and vascular cell functions. Various mechanosensory mechanisms have been suggested to mediate the effects of exercise on vascular function. The effects of repeated acute physical activities with low intensity may produce minor changes that may not be noticeable in clinical trials. However, frequent repetitions of single sessions may result in lasting adaptations (chronic effect) [109]. Therefore, even a small quantitative effect should not discourage us from adopting an active lifestyle and starting a structured exercise program. Surely, the combination of lipid interventions, including diet, medication and appropriate exercise training would prove beneficial and facilitate the optimization of individualized medical management for lipid disorders.

Future studies focusing on exercise-related improvements in HDL function should be based on standardized methodologies and require better characterization of patients from diverse populations since medications, diet and concomitant diseases may affect the final result. Polymorphisms in some genes may also affect the impact of exercise on HDL-C quality and quantity.

## Figures and Tables

**Table 1:** The summary of described studies concerning the impact of aerobic exercise on HDL levels.

Type of Exercise	Studied Population	Intensity	Duration	Additional Benefits	Ref.
On a cycle ergometer (universal aerobicycle);	200 healthy and sedentary white men (79 fathers and 121 sons) with baseline: (1) Low TG and high HDL cholesterol (normolipidemia); (2) Low TG and low HDL cholesterol (isolated low HDL cholesterol); (3) High TG and high HDL cholesterol (isolated high TGs); and (4) High TGs and low HDL cholesterol (high TG/low HDL cholesterol).	2 weeks at an HR associated with 55% VO_2_max for 30 min increased every 2 weeks until the 14th week to HR associated with 75% of their initial VO_2_max for 50 min and maintained for the next 6 weeks.	60 sessions within 21 weeks	- Lack of beneficial HDL increase in (2) group - Significant increase in HDL cholesterol and apoA-I levels (4.9% and 3.7%, respectively, *p* < 0.005) in group (4). - Increase in HDL-C in men with high TG/low HDL cholesterol mostly resulted from the rise in HDL2 cholesterol levels. - Decreased plasma TGs (≈−15.0%, *p* < 0.005) in group (3) - Significantly reduced apolipoprotein B levels at the end of the study (−6.0%, *p* < 0.005) in group (4). - Exercise-induced change in abdominal subcutaneous adipose tissue (10.6%, *p* < 0.01) strongly correlated with increase in plasma HDL cholesterol in group (4). - Significant reduction (9.0%) in the ratio of total/HDL-C in group (4) compared with other groups.	[9]
Various	4039 men and women (30–60 years) completed the study.	High-intensity intervention physical activity groups: <2 h/week, 2–3.9 h/week, 4–6.9 h/week and ≥7 h/week.	Follow-up: 5 years	- Change in serum lipids significantly associated with change in physical activity level. - Change in physical activity associated with HDL level in men only (*p* = 0.001)	[101]
Supervised endurance exercise training without weight loss Walking, jogging and stationary cycling	17 men aged 26–49 years with: (1) initially low HDL-C levels (<40 mg/dL, N = 7); (2) Normal (>44 mg/dL, N = 10) HDL-C levels on a controlled diet.	Four supervised exercise sessions/week at 60–80% of the subjects’ measured maximal HR.	12 months	- Higher increase in total HDL (5.1+/−2.8 versus 1.9+/−4.2 mg/dL, *p* = 0.08) and HDL2 (3.8+/−2.9 versus 0.4+/−1.1 mg/dL, *p* = 0.01) in group (2) - Increase in HDL2 in (2) - Decreased catabolic rates for HDL apolipoproteins by 7–14% and increase in biological half-lives by 10–15% in group (2) - HDL apolipoprotein synthetic rates were not affected by exercise training - LPLA significantly increased (by 27%, *p* < 0.01) only in group 2. - Decreased HTGLA (by ~16%, *p* = 0.06) only in group 2. - Rate of plasma triglycerides clearance increased by 14%, and apolipoprotein B levels decreased 16% (*p* < 0.01) only in group (2).	[102]
Calisthenics, walking, jogging or running	Overweight men aged 30–59 years randomized to: (1) Running (N = 46); (2) Caloric restriction (N = 42); (3) Remain sedentary, non-dieting controls (N = 42).	At the beginning, exercise for 25 min three times/week at 60% to 80% of maximal HR increased to 40 to 50 min 4 days or more per week.	l-year study	- Highest increase in HDL and HDL2 in men with normal-to-high baseline HDL from group (1), absolute increases in HDL (mg/dL) were greatest in and least in men with low baseline HDL. - Relative increases in HDL cholesterol in the low (7.1% ± 6.l%), intermediate (12.4% ± 3.9%), and normal-to-high men (13.2% ± 4.0%) were observed. - Significantly higher increase in HDL3-C concentrations in men with low HDL compared with normal-to-high HDL at - Increase in HDL2 cholesterol was nearly 4× greater in men with normal-to-high HDL compared with those with low HDL at entry. - Also, diet-related weight loss significantly increased HDL cholesterol, HDL2 cholesterol, and HDL2-mass after 1 year, respective of baseline HDL-C level.	[103]
A maximal-velocity incremented continuous treadmill run (Telineyhtymä, Kotka, Finland) at 1 slope until exhaustion	24 male national top-level endurance runners; 12 middle-distance runners; and 12 marathon runners.	High intensity.	Training and competing in middle and long distance running regularly for more than 5 years.	Immediate effects - Significantly increased oxHDL lipids by 24% (*p* < 0.01). - Considerably increased ratio of oxHDL lipids to oxLDL lipids by 55% - Significantly reduced oxLDL lipids levels (by 19%; *p* < 0.001) - Significantly increased serum malondialdehyde by 54% (*p* < 0.001) and TRAP by 29% (*p* < 0.01), After the 90 min recovery - oxHDL lipids concentration lowered to the pre-exercise level, - oxLDL lipids remained reduced below pre-exercise values (*p* < 0.001).	[104]
Cycling	20 recreationally active males allocated to: (1) Energy-deficit diet; (2) control group.		High-intensity cycling at 90% VO_2_peak at a constant workload for 8 min, 2 h after breakfast, every second day/10 days	- Decreased TG on D6-10 compared to baseline (*p* < 0.01) in group (1) - Increased LDL, TC on D2-10 vs. baseline) in group (1) - Progressively increased HDL on D6-10 (*p* = 0.01) in group (1) - No changes in TG, TC and HDL, and reduced LDL on D8-10 (*p* = 0.01) in group (2).	[107]
Aquatic exercise	Meta-analysis of 11 articles on 12 studies: (1) Exercise group (n = 208); (2) control group (n = 185).	Exercise session ranged from 15 to 60 min. The frequency of exercise was one to four sessions per week.	Duration of intervention ranged from 6 to 24 weeks (median: 12 weeks)	- Significantly decreased pooled net changes in HDL-C, LDL-C, and TC after aquatic endurance exercise (HDL-C, 4.6 mg/dL; LDL-C, −10.1 mg/dL; TC, −8.5 mg/dL) - Lack of significant changes in the pooled net changes in HDL-C, LDL-C, TC, and TG in trials related to swimming - Significantly improved pooled net changes in HDL-C, LDL-C, and TC in trials involving only females - Significantly improved pooled net changes in HDL-C, TC, and TG in trials enrolling subjects with a mean age < 60 years. - Significantly improved pooled net changes in HDL-C, LDL-C, TC, and TG in trials limited to those with dyslipidemia.	[111]
Running	36 untrained men (mean age, 31 years) assigned to: (1) Intense interval running (n = 8); (2) Heavy-resistance strength training group (n = 8); (3) Prolonged moderate-intensity continuous running (n = 9); (4) No physical training (n = 11).	(1) Intense training; (2) Strength training; (3) Moderate-intensity training.	12 weeks	- Lack of significant changes in HDL, LDL, and TC and TC:HDL ratio in (1) group - Significantly decreased TC:HDL ratio in (3) group; ratio lower than in (2) group after the 12 wk. of training	[112]
Walking–jogging, stationary cycles and treadmills	120 woman and 149 men who were sedentary, aged 50–56, free of CAD and not treated for hypertension or hyperlipidemia assigned to: (1) Higher-intensity, group-based exercise training; (2) Higher-intensity, home-based exercise training; (3) Lower-intensity, home-based exercise training; (4) A 1-year delayed treatment control (exercise training program during the second year).	(1,2) Exercise intensity gradually increased over the initial 6-week period to 73% to 88% of the peak heart rate; (3) Exercise intensity based on 60% to 73% of heart rate.	(1,2) 60 min training, 3 trainings/week (3) five 30-min exercise sessions per week	- Lack of significant increases in HDL-C during year 1 - Small but significant increase in HDL by the end of year 2 in (2) and (3) groups vs. baseline (*p* < 0 0.01). - In older individuals, time required to achieve HDL-C change after initiating a regular moderate-intensity training may be longer than that for younger populations.	[114]
Walking, running, cycling and spinning+	1567 older adults (790 (50.4%) women) assigned to: (1) Control (n = 412); (2) HIIT (n = 142); (3) MICT (n = 119).	(2) Two weekly sessions of high-intensity training (10 min warmup followed by 4 × 4 min intervals at ~90% of peak heart rate); (3) Two weekly sessions of moderate-intensity training (50 min of continuous work at ∼70% of peak heart rate).	5 years	- No changes in HDL-C after 1 year in all groups - In men, significantly improved HDL-c by 3.8% in (1) group and by 6.2% in (2) group after 3 years - Significantly reduced HDL-C concentration after 5 years in group (1) and (3), for both men and women. - In men, HDL-C was reduced 6.9% (*p* < 0.0001) in (1) group, by 7.8% (*p* = 0.001) in (3) group and by 1.2% (*p* = 0.31) in (3) group.	[116]
Walking and exercises	116 initially sedentary men and 119 women randomly assigned to: (1) 6-month lifestyle physical activity counselling intervention; or (2) 6-month gymnasium-based structured program.	(1) At least 30 min of moderate-intensity physical activity on most days of the week; (2) Traditional exercise with an intensity of 50–85% of maximal aerobic power and session durations of of 20–60 min for 3 to 5 days a week.	6 months	- Significant reductions in TC, TC/HDL-C ratio, DBP, and percentage of body fat in both groups - (1) vs. (2) group mean changes in TC: −0.2 vs. −0.3 mmol/L, TC/HDL-C ratio: −3.2 vs. −1.8 mmHg, and body fat %: −1.4 vs. −1.7%	[117]
Training on a Nautilus machine Cycling ergometry, rowing ergometry and treadmill walking/jogging	48 sedentary, healthy women (mean age, 20.4 years) assigned to: (1) Control group (n = 12); (2) Aerobic training (n = 12); (3) Resistance training (n =12); (4) Cross training (combined aerobic and resistance training, n = 12).	(2) Cycling ergometry, rowing ergometry, treadmill walking/jogging three times/week (10 min warmup, 30 min exercise at 70–75% HRmax and 10 min cool-down); (3) Training on Nautilus machine 3 times/week, 2–3 sets of 8–10 repetitions at 60–70% of established RM; (4) Combination of the above.	16 weeks of training, 6 weeks of detraining	- significantly reduced blood TG concentrations (*p* < 0.05) and significantly increased blood HDL-C concentrations after 16 weeks of training in group (2) - decreased percentage body fat by 13% (*p* < 0.05) after 16 weeks in group (2) - All effects disappeared after 6 weeks of detraining in group (2) - Lack of significant changes in TC, TG, LDL-C and HDL-C concentrations during the study in (3), (4) and (1) groups.	[118]
	111 sedentary, overweight men and women with mild-to-moderate dyslipidemia assigned to: (1) Control group; (2) High-volume high-intensity exercise; (3) Low-volume high-intensity exercise; (4) Low-volume moderate-intensity exercise.	(2) Caloric equivalent of jogging 20 mi (32.0 km)/week at 65 to 80% of VO_2_max; (3) Equivalent of jogging 12 mi (19.2 km)/week at 65 to 80% of VO_2_max; (4) Equivalent of walking 12 mi per week at 40% to 55% of VO_2_max).	6 months (1) 8 months (2)	- No significant effect on TC or LDL-C concentrations - Significantly reduced concentrations of LDL and small LDL particles and increased the average size of LDL particles in group (2). - Beneficial effect (*p* < 0.0167) on the HDL-C concentration in (2) group. - Improvement in TG concentration in (2), (3) and (4) groups (*p* = 0.006, *p* = 0.07, and *p* < 0.001, respectively), concentration of VLDL TG (*p* = 0.004, *p* = 0.04, and *p* < 0.001, respectively), concentration of large VLDL particles (*p* = 0.05, *p* = 0.13, and *p* < 0.001, respectively), and size of VLDL particles (*p* = 0.06, *p* = 0.005, and *p* < 0.001, respectively).	[119]
Exercises on treadmill; land-based or aquatically based treadmill	Overweight/obese men (n = 10) and women (n = 8).	Acute exercise session on a treadmill (70% VO_2_max, 400 kcal energy expenditure); Endurance exercise training (land-based or aquatic-based treadmill): 3 sessions/week, progressing to 500 kcal/session.	12 weeks	- Increased HDL and HDL2b-C concentrations (+3.7 and +2.4 mg/dL, *p* < 0.05) and particle numbers (+588 and +206 nmol/L, *p* < 0.05) in men. - In women, no change in total HDL-C - In women, shift in subfractions from HDL3-C (−3.2, *p* < 0.01) to HDL2b-C (+3.5, *p* < 0.05) and HDL2a-C (+2.2 mg/dL, *p* < 0.05), with increased HDL2b particle number (+313 nmol/L, *p* < 0.05). - Reduced LDL3 concentration and particle number in women (−1.6 mg/dL and −16 nmol/L, *p* < 0.05). - Acute exercise reduced TC: HDL-C ratio in men (−0.16, *p* < 0.01).	[22]
Various	Meta-analysis of data of 1555 adults from 6 studies encompassing 10 distinct exercise programs: APOE (N = 106), DREW (N = 385), GERS (N = 79), HERITAGE (N = 715), STRRIDE I (N = 168) and II (N = 102).	Various.	Various	- Regular exercise significantly decreases concentration of large VLDL-P, small LDL-P, and medium HDL-P and mean VLDL-P size, - Regular exercise significantly increases the concentration of large LDL-P and large HDL-P and mean LDL-P size.	[91]
Various	98 subjects (38 females, 60 males), aged 30–65 years with at least one CAD risk factor assigned to four groups: (1) Performance ≤99% at baseline, performance gain ≤7.9%; (2) Performance ≤99% at baseline, performance gain >7.9%; (3) Performance >100% at baseline, performance gain ≤7.9%; (4) Performance >100% at baseline, performance gain >7.9%.	At least 75 min/week of vigorous or 150 min/week of moderate-intensity endurance training.	8 months	- Significant increase in HDL-C from 52 ± 13 to 57 ± 16 mg/dL (*p* = 0.004) in group (2); - Insignificant increase in groups (1), (3) and (4) - Significant increase in apoA-I from 146 ± 24 to 160 ± 36 mg/dL (*p* = 0.002) in group (2), (3) (*p* = 0.025) and group (4) (*p* = 0.040). - No considerable changes in SAA in group (2) and (3). - SAA levels in group (1) increased after 3 months, but returned to baseline values after 8 months - In group (2), SAA decreased first 4 months of intervention	[39]
Bicycle ergometers	30 sedentary subjects (20 with and 10 without MS).	Moderate-intensity exercise training on bicycle ergometers.	3 months	- Decreased plasma TG - No impact on LDL-C or HDL-C levels - Increased HDL subfractions’ antioxidative capacity and PON-1 activity. - Compositional changes in the smallest HDL subfractions associated with increased free cholesterol and cholesterol ester transfers to HDL	[92]
Running, throwing hammer, wrestling and weightlifting	National-class male athletes: representatives in running (1500 m middle distance, n = 10), throwing (hammer, n = 10), wrestling (n = 10) and weightlifting (n = 8). Age- and gender-matched sedentary reference subjects (n = 14).	Athletes: exercising at least 8 h per day; Reference: regular exercise with moderate intensity less than 1 h/week.	At least 6 years,	- Significantly lower TC and TG concentrations in athletes’ sera (regardless of the type of sport and BMI) compared with reference group - Significantly higher HDL-C levels in runners and wrestlers (constituting 35–37% of TC), then in throwers and lifters (31% of TC), and reference (24% of TC). - Highest antioxidant activity of HDL3 in runners, followed by wrestlers and throwers while lifters showed the weakest antioxidant activity - Higher LCAT activity of HDL3 from athletes compared to reference group - Highest activity in runners and wrestlers - Higher PON activity of HDL3 (antioxidant activity) in athletes, especially wrestlers and runners) compared to reference HDL3 Highest PON-1 protein expression in runners (BI = 3.0), followed by wrestlers (BI = 2.8). - Highest level of HDL2 apoA-I in wrestler group - Highest level of HDL3 apoA-I in runners compared to the other athletes and reference group, then in wrestler group - In wrestlers, HDL2 of largest in size, followed by the runners. - Among athletes, the lifters had the smallest HDL2 particle	[140]
Walk/run training	35 patients with MS assigned to: (1) Control group (n = 12); (2) Exercise group (n = 27).	10-week walk/run training program.	10 weeks	- Lack of impact of training on lipoprotein level in and cholesterol efflux capacity of circulating HDL - Walk/run training increased paraoxonase-1 (PON1) activity and decreased the levels of malondialdehyde in serum or isolated HDL - Isolated HDL3 protected endothelial cells TNF-α-induced injury, decreased MCP-1 levels in media and VCAM-1. - Isolated HDL3 inhibited TNF-α-induced monocyte adhesion to endothelial cells and increased NO production by activating eNOS.	[141]
Aerobic dance, running, skipping, and stepping Bands and free weights; Squats, lunges, bicep curls, push-ups and shoulder presses	32 obese black South African women aged 20–35 years assigned to: (1) Exercise (combined aerobic and resistance exercise) (n = 20); or (2) Control (<1 session of <20 min per week) (n = 15).	Aerobic and resistance exercise training of moderate–vigorous intensity (75–80% peak heart rate, HR peak) for 40 to 60 min 4 days/week supervised by a trained exercise physiologist. Prescribed intensity of 60% to 70% HRpeak.	12-weeks	- Decrease in body mass index (*p* = 0.010), PON activity, PAF-AH serum expression (*p* = 0.002), and the distribution of small HDL subclasses *p* = 0.004) compared to controls. - Lack of impact on HDL cellular cholesterol efflux capacity and anti-inflammatory function.	[24]
Walking or Nordic walking, jogging, cycling, swimming, skiing, aerobics or other gymnastic exercise	161 sedentary women aged 43–63 years with no current use of hormone therapy assigned to: (1) Exercise group; (2) Control group.	Unsupervised aerobic training 4 times/week, 50 min of the exercise each time, 64–80% of the maximal HR.	6 months	- Increased concentration of ox-HDL in (1) group (by 5%) and decreased in (2) group (by 2%) (*p* = 0.003). - Increased ratio of ox-HDL:HDL-C (by 5%) in (1) group and decreased (by 1.5%) in (2) group (*p* = 0.036). - No changes in CETP concentrations and adiponectin - Decreased serum TG triglycerides (by 6%) in (1) group (*p* = 0.051). - Increase in ox-HDL was not associated with changes in the levels of CETP or adiponectin.	[139]

CETP, cholesteryl ester transfer protein; DBP, diastolic blood pressure; eNOS, endothelial nitric oxide synthase, LCAT, lecithin: cholesterol acyltransferase; HTGLA, hepatic triglyceride lipase; HR, heart rate; LPLA, lipoprotein lipase; MCP-1, monocyte chemotactic protein-1; MS, metabolic syndrome; NO, nitric oxide; PON-1, paraoxonase-1; RM, repetition maximum; TNF-a, tumor necrosis factor-a; TRAP, serum antioxidant potential; VCAM-1, vascular cell adhesion molecule-1; VO_2_max, peak oxygen consumption.

## Data Availability

Not applicable.
